# Peptide Location Fingerprinting Reveals Tissue Region-Specific Differences in Protein Structures in an Ageing Human Organ

**DOI:** 10.3390/ijms221910408

**Published:** 2021-09-27

**Authors:** Alexander Eckersley, Matiss Ozols, Peikai Chen, Vivian Tam, Judith A. Hoyland, Andrew Trafford, Danny Chan, Michael J. Sherratt

**Affiliations:** 1 Division of Cell Matrix Biology & Regenerative Medicine, Faculty of Biology, Medicine and Health, School of Biological Sciences, The University of Manchester, Manchester M13 9PT, UK; matiss.ozols@manchester.ac.uk (M.O.); judith.a.hoyland@manchester.ac.uk (J.A.H.); 2 Department of Human Genetics, Wellcome Sanger Institute, Genome Campus, Hinxton CB10 1SA, UK; 3 School of Biomedical Sciences, The University of Hong Kong, Hong Kong, China; pkchen@hku-szh.org (P.C.); vivtam@hku.hk (V.T.); chand@hku.hk (D.C.); 4 Department of Orthopaedics and Traumatology, The University of Hong Kong-Shenzhen Hospital (HKU-SZH), Shenzhen 518053, China; 5 NIHR Manchester Biomedical Research Centre, Central Manchester Foundation Trust, Manchester Academic Health Science Centre, Manchester M13 9PL, UK; 6 Manchester Academic Health Science Centre, Division of Cardiovascular Sciences, Faculty of Biology, Medicine and Health, The University of Manchester, Manchester M13 9PL, UK; Andrew.W.Trafford@manchester.ac.uk

**Keywords:** proteomics, peptide location fingerprinting, extracellular matrix, ageing, intervertebral disc, spine, mass spectrometry

## Abstract

In ageing tissues, long-lived extracellular matrix (ECM) proteins are susceptible to the accumulation of structural damage due to diverse mechanisms including glycation, oxidation and protease cleavage. Peptide location fingerprinting (PLF) is a new mass spectrometry (MS) analysis technique capable of identifying proteins exhibiting structural differences in complex proteomes. PLF applied to published young and aged intervertebral disc (IVD) MS datasets (posterior, lateral and anterior regions of the annulus fibrosus) identified 268 proteins with age-associated structural differences. For several ECM assemblies (collagens I, II and V and aggrecan), these differences were markedly conserved between degeneration-prone (posterior and lateral) and -resistant (anterior) regions. Significant differences in peptide yields, observed within collagen I α2, collagen II α1 and collagen V α1, were located within their triple-helical regions and/or cleaved C-terminal propeptides, indicating potential accumulation of damage and impaired maintenance. Several proteins (collagen V α1, collagen II α1 and aggrecan) also exhibited tissue region (lateral)-specific differences in structure between aged and young samples, suggesting that some ageing mechanisms may act locally within tissues. This study not only reveals possible age-associated differences in ECM protein structures which are tissue-region specific, but also highlights the ability of PLF as a proteomic tool to aid in biomarker discovery.

## 1. Introduction

Intervertebral discs (IVDs) are fibrocartilaginous cushions residing between vertebrae, which confer flexibility and stability to the spinal column and function as effective shock absorbers over an individual’s lifetime [1]. Age-associated degeneration of the IVD in the lower lumbar spine is the leading cause of lower back pain within an ageing population [2], imposing major socioeconomic and healthcare burdens.

IVDs (located between the endplates of vertebrae) consist of two extracellular matrix (ECM)-rich tissue compartments: (i) a central gel-like nucleus pulposus (NP), composed predominantly of the chondroitin sulphate proteoglycans (CSPGs) aggrecan and versican, and a relatively disordered network of collagen II and (ii) an encircling annulus fibrosus (AF) consisting of highly ordered, concentric rings (lamellae) of parallel collagen I and elastic fibres arranged in a crisscross pattern [3,4]. These compartments are large, avascular and subject to mechanical loads, creating a challenging environment for tissue repair and regeneration (mediated mainly by a small population of chondrocyte-like cells in the NP and fibroblast-like cells in the AF [5]). Crucially, regional differences in ECM composition exist within the AF. ECM components within the inner AF reflect the interface between the NP and outer AF, being comprised of not only collagen I fibrils but also CSPGs and collagen II fibrils [4,6].

Clinically, the onset and progression of IVD ageing and degeneration is characterised typically by a gradual reduction of hydration, pressure and height of the NP and the formation of tears in AF lamellae making the disc more prone to bulging, protrusion or herniation [7]. This structural damage to the AF, and hence susceptibility to failure, is profoundly regional, with degeneration mainly localised in the posterior-lateral portion of the IVD [8,9]. The dynamic alteration in ECM composition and abundance likely underpins this regional progression of IVD ageing and degeneration, with decreasing levels of CSPGs in both the NP and the AF and increasing levels of collagen I fibres, leading to a fibrotic, dehydrated phenotype [4,10,11,12,13]. Additionally, we previously showed that the protein composition of the inner AF becomes gradually more similar to that of the outer AF with ageing [13]. It is clear, therefore, that understanding the mechanisms and consequences of ECM remodelling is key to defining and potentially reversing IVD degeneration and ageing.

ECM assemblies and components can be long-lived, with biological half-lives measured in years or decades. For example, lung elastic fibres and collagens in cartilaginous tissues remain present throughout the life of an individual [14,15]. Consequently, ECM assemblies residing in the IVD (such as collagen I in the AF [16] and aggrecan in the NP [17]) are susceptible to the progressive accumulation of damage to both their molecular structures and networks over time, leading to loss of functions of many diverse ECM components. The potential causative mechanisms of this remodelling include the chronic exposure of ECM proteins to elevated protease-mediated cleavage [18], cross-linking by sugars leading to advanced glycation end products (AGEs) [19], mechanical fragmentation [20] and oxidation by reactive oxygen species (ROS) [21]. These processes may lead to a spectrum of modifications to protein higher order structures that are challenging to detect. Although several proteomics studies have characterised IVD ageing and degeneration through changes in protein presence and abundance alone [12,13,22,23,24], the identification of ECM proteins susceptible to damage modifications to their structure is also crucial both for the discovery of novel ageing biomarker candidates and for deciphering the underlying mechanisms involved.

Peptide location fingerprinting (PLF) is a new computational technique capable of identifying proteins exhibiting structural differences in mass spectrometry (MS) datasets derived from complex biological samples regardless of the causal mechanisms [25]. In standard proteomics, proteins are exposed to enzymes (e.g., trypsin), generating peptides whose sequence identities and relative abundances are then measured by liquid chromatography tandem MS (LC–MS/MS). PLF relies on the fact that, for a given protein, the pattern of peptides detected by LC–MS/MS following protease digestion is determined by its higher order structure and reflects its solubility, stability and enzyme susceptibility. Therefore, differences in protein structure, due to either the accumulation of damage (via ageing and disease), changes in interaction states with other protein or differential synthesis may be detected by PLF [25,26,27]. This is particularly true for the highly insoluble ECM proteins that exist within tightly bound networks. In standard LC–MS/MS, peptides are mapped to proteins, enabling the quantification of protein abundance. PLF maps and quantifies peptides (by spectral counting) within specific protein regions instead, enabling the detection of statistical differences along the primary structure (peptide yield patterns) [25,26]. Previously, we used PLF to reproducibly detect UVR-induced damage within the structures of photosensitive skin ECM proteins in vitro [26] and tissue-specific differences in eye (ciliary zonule) and skin fibrillin-1 [27]. Crucially, and most recently, we have developed and employed an online webtool (MPLF) to identify potential proteins with structural differences between photoaged and photo-protected skin and young and aged tendon [25]. Comparisons with conventional protein quantification also revealed that PLF identifies significant differences, which are unique to the methodology, particularly in ECM proteins, demonstrating that modifications to protein structure may not correlate with protein abundance in damage-susceptible long-lived proteins [25].

Recently, we have also published DIPPER, a detailed spatiotemporal proteomic analysis of the ageing and degenerating human IVD, which included high-resolution label-free LC–MS/MS performed on 11 separate regions of discs sourced from two male cadaveric lumbar spines, one young (16 years old) and one aged (59 years old) [13]. This study revealed novel, region-specific differences in ECM proteome (matrisome) composition and abundance, which demonstrated a dynamic shift along the lateral and anteroposterior axes, with a rapid decline of NP proteins and an increase in AF proteins in the aged inner AF. Although this proteomic study was highly detailed, modification-related differences to ECM protein structures were not interrogated. Since degeneration in the ageing disc is profoundly regional [8,9], and PLF can be applied post hoc to historical LC–MS/MS data (as shown for ageing tendon [25]), further analysis of the DIPPER dataset presents us with a timely opportunity to test: (i) the hypothesis that protein structures (as indicated by peptide yield pattern) are spatially and potentially age-dependent in the IVD and (ii) the ability of PLF to detect protein structural differences within localised tissue regions.

**Figure 1:**
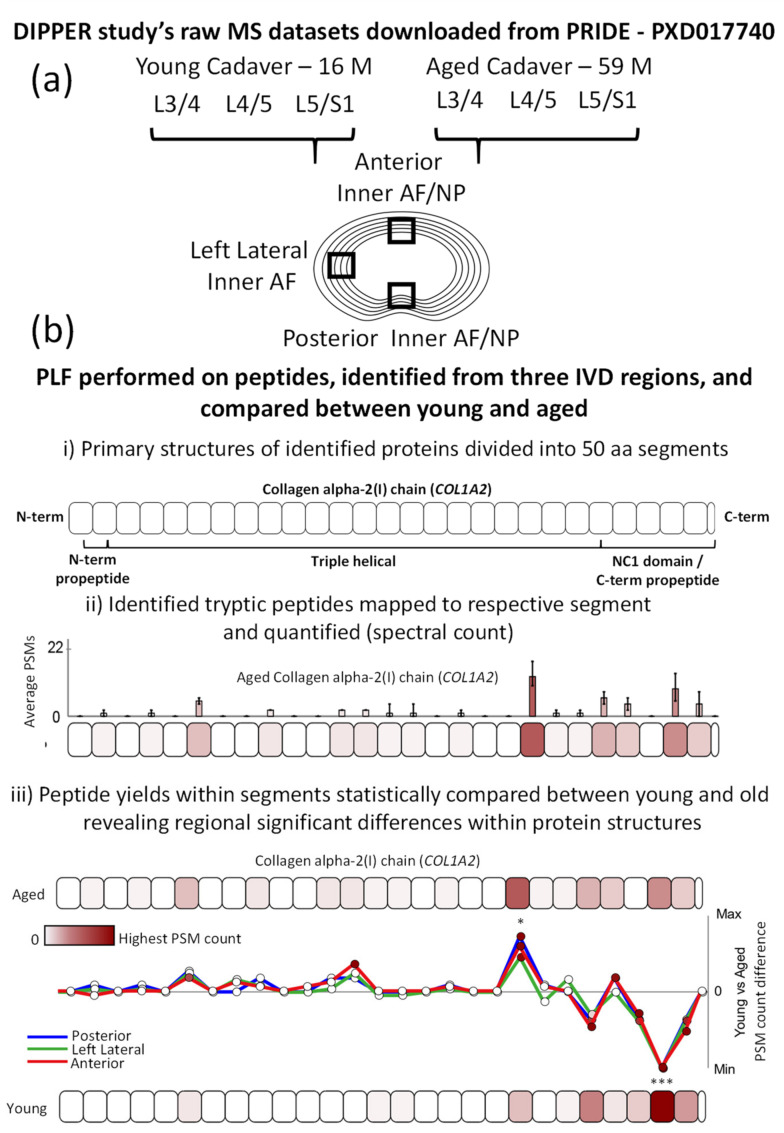
Experimental design for the application of PLF to three distinct regions of young and aged IVDs. (**a**) Raw LC–MS/MS datasets (from two males, one young [16 years old] and one aged [59 years old]), corresponding to the posterior inner AF/NP, left lateral inner AF and anterior inner AF/NP regions of the L3/4, L4/5 and L5/S1 lower lumbar IVDs were downloaded from the PRIDE repository (PXD017740) [13]. (**b**) After MS/MS peptide identification, the MPLF webtool [25] was used to perform PLF on the identified peptides and compare peptide yields across protein structures between young and aged discs—three vs. three discs for anterior and left lateral datasets and three vs. two discs for posterior datasets (the young anterior L4/5 disc was omitted from the analysis due to the insufficient number of identified peptides for PLF analysis). As previously described [25], ((**b**), i) proteins were divided into 50-amino acid (aa)-sized segments; the collagen I α2 chain (*COL1A2*) is shown here as an example. ((**b**), ii) Peptides were mapped to their respective segments, summed, normalised between aged and young groups based on the median whole protein spectrum count and averaged for each group (average peptide spectrum match [PSM] count per segment). ((**b**), iii) Peptide yields in each segment were statistically compared between young and aged IVDs using an unpaired, Bonferroni-corrected, repeated-measures ANOVA (* *p* ≤ 0.05; *** *p* ≤ 0.001). To visualise these differences along the protein structure, average PSM counts per segment in the young group were subtracted from those in the aged one and compared between posterior, left lateral and anterior IVD regions.

As the inner AF appeared particularly susceptible to matrisomal changes [13], we applied PLF to LC–MS/MS datasets corresponding to the inner AFs of three distinct IVD regions (posterior, left lateral and anterior; Figure 1a). This enabled the detection of structural differences in ECM proteins between aged and young IVDs, which could be further compared between degeneration-prone (posterior and lateral) and -resistant (anterior) regions of the IVD (Figure 1b), revealing potentially age-affected proteins with tissue region-specific peptide yield patterns. Please note, in the following text and figures, proteins are commonly referred to by their gene name (indicated by italics).

## 2. Results and Discussion

### 2.1. Peptide Location Fingerprinting Reveals Proteins with Region-Specific Differences between Aged and Young IVDs

**Figure 2:**
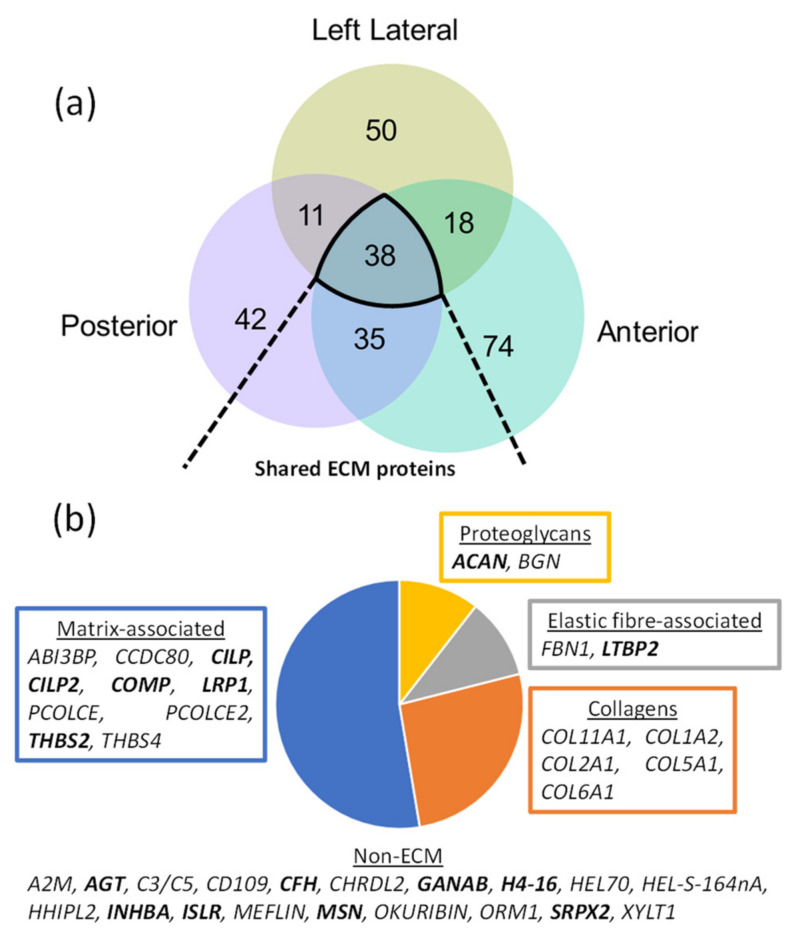
ECM-associated proteins make up half of the proteins identified with significant, age-associated structural differences in all three IVD regions. PLF revealed 126 proteins in posterior (Table S4), 117 in left lateral (Table S5) and 165 in anterior (Table S6) IVD regions with significant differences in peptide yield between aged and young IVDs, across their structures. (**a**) Of these, 38 were shared between the three regions. (**b**) Classification of these shared proteins revealed ECM as the major class, which included several ECM-associated proteins, collagens, two elastic fibre-specific proteins and two proteoglycans. The affected proteins which were uniquely discovered by PLF and not by label-free quantification of abundance in the DIPPER study [13] (Figure S3) are highlighted in bold.

MS/MS peptide searches identified 260 posterior, 391 left lateral and 303 anterior inner AF-specific proteins in both young and aged groups (Figure S1; peptide reports: Tables S1–S3). Principal component analyses of peptide spectral counts demonstrated clear separations of data between young and aged discs for all three IVD regions tested (Figure S2), reflecting a global compositional difference in ageing similar to that showcased previously in the DIPPER study [13]. PLF analysis identified a combined total of 268 proteins (across tissue regions) with significant differences in peptide yield patterns along their primary structures (Figure 2a; full PLF analysis reports: Tables S4–S6). When compared to the proteins identified in the DIPPER study with significant differences in relative abundance between aged and young samples (by label-free quantification) [13], PLF identified 119 proteins that were unique to this approach, with significant differences in protein structure but not abundance (Figure S3).

Of the 268 proteins identified with age-associated structural differences, 38 were shared between posterior, lateral and anterior inner AF regions (Figure 2a). Classification of these proteins into ECM-specific protein classes highlighted several matrix-associated proteins, including the major structural collagens of the AF (collagen I [*COL1A2*] and V [*COL5A1*]) and NP (collagen II [*COL2A1*] and VI [*COL6A1*]), elastic fibre-associated proteins (fibrillin-1 [*FBN1*] and LTBP2 [*LTBP2*]) and proteoglycans such as aggrecan (*ACAN*] as key affected components (Figure 2b). Some of the identified proteins have been linked to disease, including osteoarthritis-associated [28,29] cartilage intermediate layer proteins 1 and 2 (*CILP* and *CILP2*), the levels of which were shown to differ significantly between degenerated and non-degenerated AFs and NPs [12], and cell-matrix adhesion-mediating thrombospondins 2 [30] and 4 [31]. The levels of thrombospondin 4 (*THBS4*) were shown previously in the DIPPER study to be significantly higher in the young inner AF than in the aged one [13]. However, to our knowledge, age-associated structural differences in disc thrombospondin have not been previously shown, although two single-nucleotide polymorphisms were found to be associated with IVD degeneration [32].

The decline of the proteoglycan aggrecan in IVD ageing due to proteolysis by MMPs and aggrecanases is well established [33]. Here, we show evidence of structural differences within the aggrecan core protein between aged and young IVDs which may relate to these mechanisms. Similarly, the degeneration of elastic fibre and collagen architectures are a hallmark of ageing in many connective tissues such as IVD, skin and aorta [34,35,36,37,38]. Our data show that, in the IVD, this process may possibly extend specifically to modification-associated changes in the protein structures of elastic fibre proteins fibrillin-1 and latent TGFβ-binding protein 2 and in multiple α-chains from collagens I, II, V, VI and XI.

To investigate whether the age-associated differences in peptide yield patterns along the primary structure of ECM proteins are consistent between regions, members of these 38 shared, significantly different proteins were examined further.

### 2.2. Age-Associated Fluctuations in Peptide Yield along the Primary Structures of COL1A2 and PCOLCE Are Markedly Conserved between Posterior, Lateral and Anterior IVD Regions

Both the collagen I α2 chain (*COL1A2*) and procollagen C-endopeptidase enhancer 1 (*PCOLCE*; *PCPE1*) were identified with age-associated modifications to their structures in all three IVD regions (Figure 2b). *COL1A2* is a major structural component of collagen I fibrils, forming a tightly bound triple helix alongside two additional α1 chains (*COL1A1*) within a monomer. The extracellular glycoprotein procollagen C-endopeptidase enhancer 1 (*PCOLCE*) binds to the C-terminal propeptide of procollagens and drives the activity of tolloid proteinases, procollagen cleavage and their subsequent assembly into fibres [39]. *PCOLCE* therefore plays a crucial role in collagen deposition and associated morbidities such as fibrosis [40].

**Figure 3:**
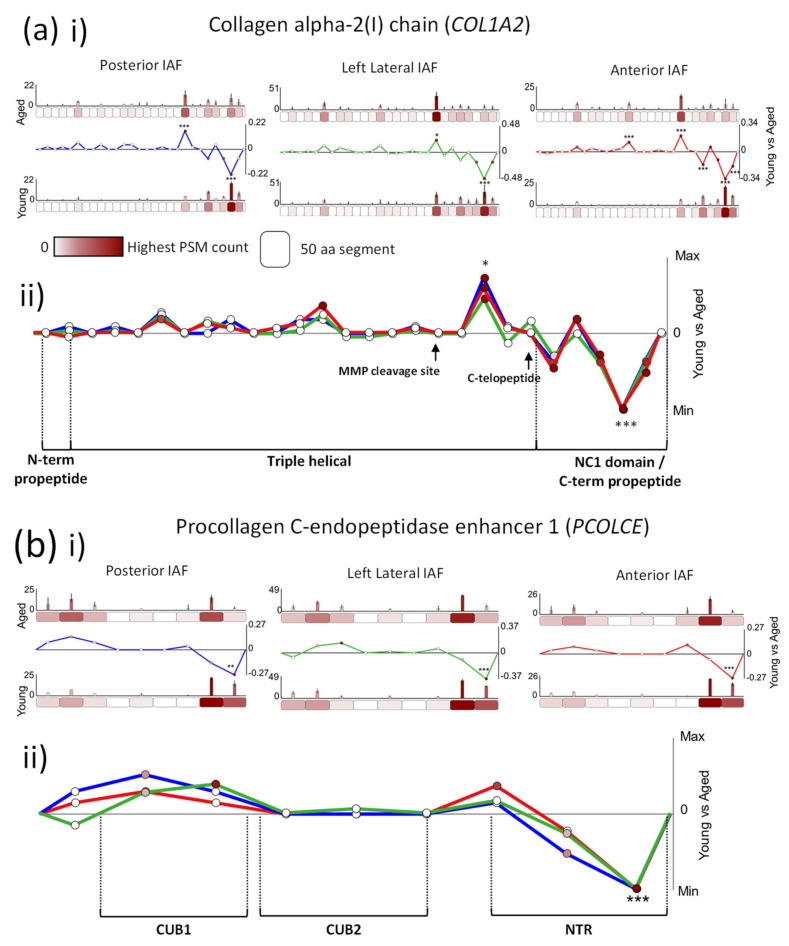
*COL1A2* and *PCOLCE* protein structures exhibit differences in peptide yield patterns between young and aged proteins, which were conserved in posterior, left lateral and anterior IVD regions. PSMs were summed in each 50-aa protein segment, normalised and averaged for young and aged groups (bar graphs = average, normalised PSMs, error bars = standard deviation). Average PSMs per segment in young were subtracted by those in aged and divided by segment aa length (50) to reveal fluctuations in peptide yield along protein structures (line graph y axes = aged–young PSM counts/segment length, normalised between tissue regions in composite graphs; * *p* ≤ 0.05; ** *p* ≤ 0.01; *** *p* ≤ 0.001, Bonferroni-corrected, repeated-measures ANOVA; black * in composite line graph = significant in all three IVD regions; functional domains, sourced from UniProt, are indicated). ((**a**), i) Several *COL1A2* segments exhibited significant differences in peptide yield between young and aged samples within the C-terminal half of the protein (blue line: posterior, green: left lateral, red: anterior). ((**a**), ii) Crucially, these differences in peptide yield along the *COL1A2* structure are markedly consistent between all the three regions, with a similarly higher peptide yield displayed in segment 20 of aged compared to young and lower peptide yield in the last three segments, nearest to the C-terminus. ((**b**), i) The last 50-aa segment on the C-terminal end of *PCOLCE* also exhibited a significantly lower peptide yield in aged compared to young for the inner AFs of all three IVD regions. ((**b**), ii) Peptide yield difference patterns along the primary structure were also similar between all the three regions, with a consistently higher peptide yield in aged compared to young measured on the N-terminal side of the protein followed by a lower yield in the last two segments near the C-terminal end.

Comparisons between posterior, lateral and anterior regions revealed notable, consistent age-associated differences in peptide yield across the protein structures of *COL1A2* and *PCOLCE*. On the C-terminal side of the *COL1A2* protein, characteristic significant increase and decrease in peptide yield pattern were observed in aged compared to young, along the primary structure for all three IVD regions (Figure 3a). Similarly, a characteristic increase in peptide yield was seen on the N-terminal side of aged *PCOLCE* compared to young *PCOLCE*, followed by a significant decrease in peptide yield on the C-terminal side (Figure 3b). This pattern was also consistent for posterior, lateral and anterior inner AF regions. While there were age-associated differences observed along the structures of both proteins, it is important to consider that peptide yields were also stable between aged and young in many protein regions (such as on the N-terminal side of *COL1A2*). This lack of difference between young and old was also consistent between IVD regions.

The significantly higher peptide yield, observed on the C-terminal end of young *COL1A2* compared to the aged protein, corresponds directly to its non-collagenous (NC1) domain (Figure 3a(ii)). These domains are cleaved prior to fibril assembly [41] by C-proteinases such as bone morphogenic protein-1/tolloid proteinase (BMP1) from fully folded procollagen, releasing a C-terminal propeptide. Since the release of this domain is critical for the formation and assembly of the collagen triple helix protomer [42], its higher detection in the young IVD suggests heightened collagen synthesis compared to the aged one. Also in support of this, the significantly lower peptide yield in aged *PCOLCE* compared to young *PCOLCE*, seen on its C-terminal end for all IVD regions, coincides directly with its netrin (NTR) domain (Figure 3b(ii)), critical for the super-stimulation of BMP1 and its cleavage of the collagen NC1 domain prior to fibril assembly [43]. This lower peptide yield in the netrin domain of aged *PCOLCE* may impact on its interaction with BMP1. It is possible that age-associated changes to *PCOLCE* structure may have resulted in masking of this protein region, which would explain its lower availability to trypsin and potentially BMP1, also impacting on collagen synthesis. Recent studies of collagen turnover in healthy tendon have shown that collagen is not repaired [44,45] but instead maintained daily, through a cycle of synthesis and degradation that is controlled by the circadian rhythm [46]. It is likely that a similar maintenance program exists in young, healthy AF (compositionally similar to tendon), and here, we show indications that this may deteriorate with age.

A second *COL1A2* protein region, located near the C-terminal end of the triple helical domain (Figure 3a(ii)), also differed significantly between aged and young samples (higher peptide yields in aged) for all three IVD regions. As this protein region is located well within the triple helix of collagen I, peptides derived from this domain are likely to be from mature collagen fibrils. In osteoporosis, fragments of the C-terminal telopeptide of collagen I α1 are frequently used as markers of collagen degradation in bone [47,48]. Although the *COL1A2* protein region seen here is located upstream of its C-terminal telopeptide (Figure 3a(ii), black arrow), a higher peptide yield in aged IVD may also be indicative of collagen fibril degradation as a consequence of damage accumulation to structure. *COL1A2* is a substrate for matrix metalloproteinases (MMPs) 1, 2, 3, 8 and 14 [49] (see interaction network Figure S4), all of which were shown to be upregulated in degenerated IVD [50]. All of these enzymes (except MMP3) preferentially cleave the α-chains of collagens I, II and III at a point approximately three-quarters of the way along the mature chain from the N-terminus [51,52]. The *COL1A2* region, which had higher peptide yields in aged than in young samples, is located just downstream of this prominent MMP cleavage site (indicated in Figure 3a(ii), black arrow) and corresponds to this degradation fragment, cleaved three-quarters away from the N-terminus. This suggests that chronic exposure to elevated endogenous MMPs, thought to be one of several ageing mechanisms driving the accumulation of damage to long-lived ECM assemblies, could potentially be playing a role in the higher peptide yield observed in the triple-helical domain of aged *COL1A2* compared to young *COL1A2*.

In addition to BMP1, *PCOLCE* also binds fibronectin [53], LTBP1, thrombospondin 1, aggrecan and biglycan [54] (and their associated glycosaminoglycans), and *COL1A2* binds *COL5A1* [55] within the wider ECM network (Figure S4). If the structural differences seen in aged *PCOLCE* and *COL1A2* compared to the young molecules (Figure 3) result in the impediment of these interactions, it could drive the deterioration of the wider proteostasis [13].

The remarkable consistency in the age-associated peptide yield patterns of *COL1A2* and *PCOLCE* structures between the posterior, lateral and anterior regions of the inner AF (Figure 3) is an interesting observation, as it indicates the possibility that ageing mechanisms may be at play tissue-wide. However, some proteins exhibited significant structural differences in peptide yield, which were entirely tissue region-specific.

### 2.3. Age-Associated Peptide Yield Patterns along the Primary Structures of the α1 Chains of Collagens II and V and Aggrecan Exhibit Regional Tissue Specificity within the IVD

**Figure 4:**
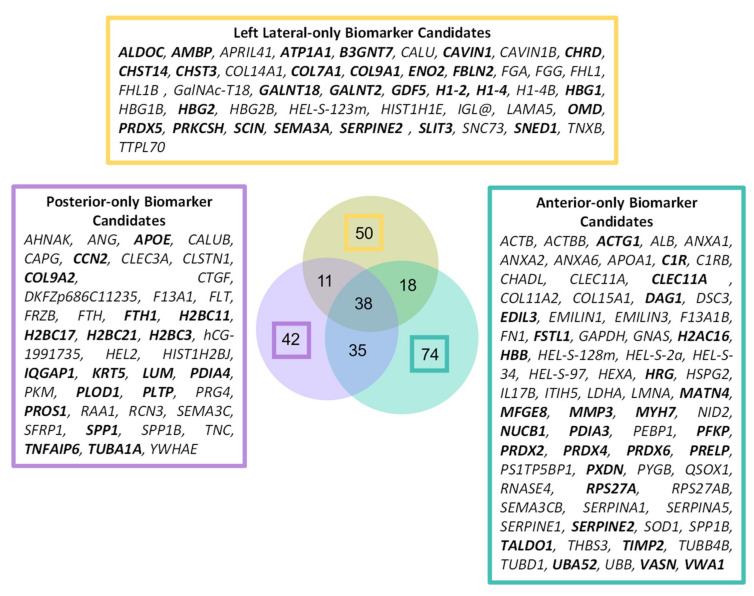
A total of 166 proteins were identified with significant, age-associated structural differences that were entirely IVD-region specific. The affected proteins which were uniquely discovered by PLF and not by label-free quantification of abundance in the DIPPER study (Figure S3) [13] are highlighted in bold.

A sub-population of IVD proteins showed significant differences in structure which were region-specific (42 for posterior, 50 for lateral and 74 for anterior) between young and aged samples (Figure 4). These proteins included several prominent ECM components such as tenascin (*TNC*) and proteoglycan 4 (*PRG4*) (exclusive to posterior), collagen XIV α1 (*COL14A1*) and osteomodulin (*OMD)* unique to left lateral) and EMILIN-1 (*EMILIN1*) and nidogen 2 (*NID2*) (exclusive to anterior) (Figure S5).

**Figure 5:**
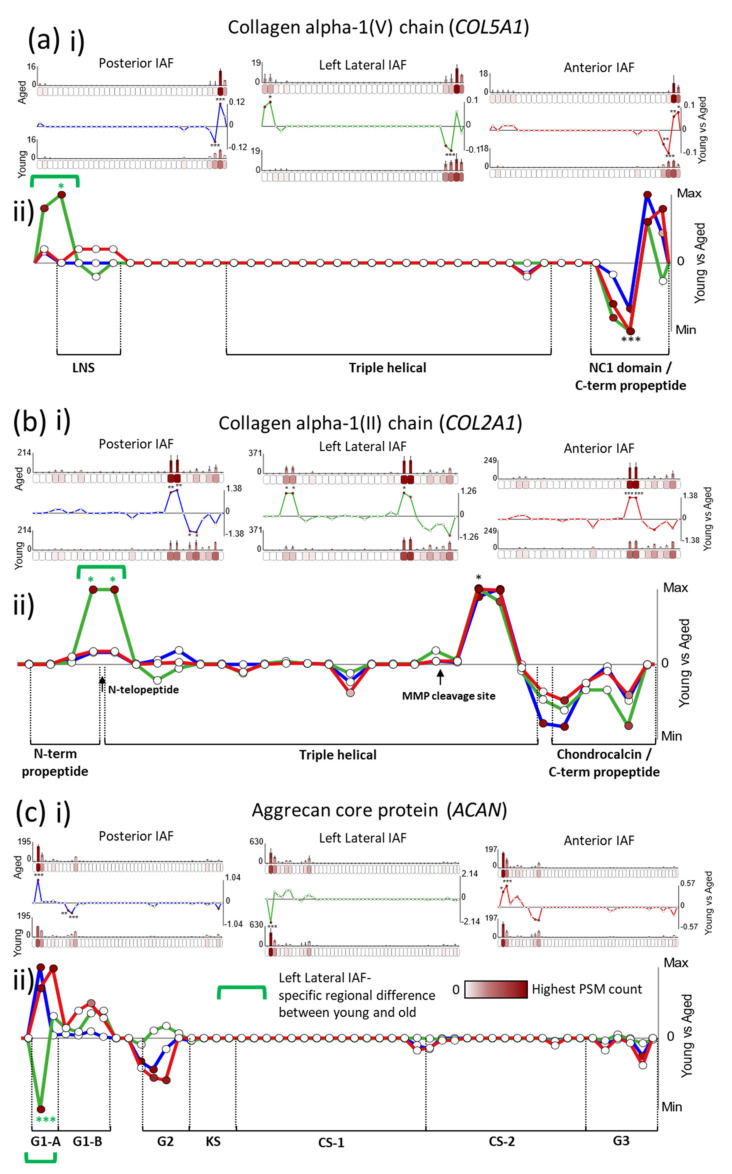
*COL5A1*, *COL2A1* and aggrecan all exhibit lateral, inner AF-specific differences in peptide yield not seen in the posterior or anterior IVD regions. PSMs were summed in each 50-aa protein segment, normalised and averaged for young and aged groups (bar graphs = average, normalised PSMs, error bars = standard deviation). Average PSMs per segment in the young group were subtracted by those in the aged one and divided by segment aa length (50) to reveal fluctuations in peptide yield along protein structures (line graph y axes = aged–young PSM counts/segment length, normalised between tissue regions in composite graphs; * *p* ≤ 0.05; ** *p* ≤ 0.01; *** *p* ≤ 0.001, Bonferroni-corrected, repeated-measures ANOVA; black * in composite line graph = significant in all three IVD regions; functional domains, sourced from UniProt, are indicated). ((**a**), i) Several segments on the C-terminal end of *COL5A1* exhibited significant differences in peptide yields between young and aged samples for all three IVD regions. However, a left lateral-specific significant difference in yield was also observed in its second segment, on the N-terminal end of the protein. ((**a**), ii) Fluctuations in *COL5A1* peptide yield on the C-terminal end follow a similar pattern in all three regions, with a consistent fall and rise of peptide yield observed in aged compared to young samples for the last four segments of its primary structure. However, a clear left lateral-specific increase in peptide yield can also be seen on the N-terminal end. ((**b**), i) *COL2A1* also had several protein segments on the C-terminal side which exhibited significant differences in peptide yield. ((**b**), ii) These displayed a characteristic rise-and-fall peptide yield pattern in aged compared to young samples, which were markedly consistent between the three IVD regions. However, once again, two *COL2A1* segments on the N-terminal side of the protein exhibited significantly higher yields for aged than for young samples that were unique to the left lateral region of the disc. ((**c**), i) Segments two and three on the N-terminal side of the aggrecan protein (ACAN) also exhibited significant differences in peptide yield between young and aged samples. ((**c**), ii) These differences were significantly higher in aged aggrecan within the posterior and anterior inner AF regions, compared to young aggrecan, but significantly lower in the left lateral region.

Importantly, some proteins even displayed differences in peptide yield between young and aged samples, which were shared between all AF regions (Figure 2b) on one side of their protein structures but region-specific on the other side, as seen for collagen V α1 (*COL5A1*), collagen II α1 (*COL2A1*) and aggrecan core protein (*ACAN*) (Figure 5). For *COL5A1*, a characteristic significant decrease in peptide yield in aged compared to young samples, followed by an increase, can be seen in the C-terminal end, along the primary structure, which remains consistent for all three IVD regions (Figure 5a). This is similar also for *COL2A1*, which exhibited the same significant rise and fall in peptide yield along its chain in all three IVD regions, on its C-terminal side (Figure 5b). However, both α1 chains of collagens V and II displayed additional N-terminal, significant increases in peptide yield in aged compared to young samples that were lateral-specific (green brackets in Figure 5a,b(ii)). Contrastingly, a significantly higher peptide yield was observed near the N-terminal end of aged aggrecan than in the young one, for both the posterior and the anterior inner AF regions (Figure 5c). However, this pattern was reversed for left lateral, with the same protein region yielding significant lower peptide counts in aged aggrecan than in young aggrecan (green bracket in Figure 5c(ii)).

The tissue region-conserved, higher peptide yields seen on the C-terminal ends of young COL5A1 and COL2A1 compared to the aged one (Figure 5a,b(ii)) also corresponded to their cleaved C-terminal propeptides, as seen for *COL1A2* (Figure 3a(ii)). This once again reflects a possible tissue-wide perturbation in collagen maintenance [46] in aged compared to young discs, for collagen II and V fibrils in addition to collagen I. Interestingly, we noticed that the C-terminal propeptides of *COL1A2* and *COL5A1* are enriched in ROS-sensitive residues [56] (Figure S6), indicating a potential role for these fragments as antioxidants, whose presence is reduced in aged IVD (Figure 3a and Figure 5a(ii)). Again in common with *COL1A2* (Figure 3a(ii)), aged *COL2A1* also exhibited an AF region-wide significant increase in peptide yield compared to young *COL2A1*, downstream to a prominent MMP cleavage site (Figure 5b(ii), black arrow) [51,52], possibly suggesting that chronic degradation of collagen II by MMPs may be an ageing mechanism at play.

The lateral-specific, significant rise in peptide yield seen on the N-terminal end of aged *COL5A1* compared to young *COL5A1* (green bracket in Figure 5a(ii)) corresponded partly to its laminin G-like (LNS) domain [57]. Although capable of matriglycan interactions [58], the function of this domain specifically within collagen V fibrils remains elusive. Here, we show clear evidence that the binding availability of the LNS domain is significantly higher exclusively in left lateral aged *COL5A1* than for all other groups (young, aged and posterior, anterior). For *COL2A1*, these aged, lateral-specific increases in peptide yield (Figure 5b) corresponded directly to the interface between the N-terminal propeptide and the triple-helical region, where the telopeptide is located. Collectively, these observations in *COL5A1* and *COL2A1* may indicate the degradation of their respective collagen V and II fibrils exclusively in the lateral regions of the AF.

The heightened peptide yield observed near the N-terminal end of aged posterior and anterior *ACAN* compared to young *ACAN*, which was reversed (lower) for the aged left lateral protein, coincides with the immunoglobulin fold of its globular G1-A domain (Figure 5c(ii)), [33]. The A subdomain in particular is critical for stabilising and enhancing the binding of its adjacent B subdomains to hyaluronan [59]. This interaction is crucial for the anchorage of aggrecan to collagen fibrils in articular cartilage [60] (Figure S4). Several ageing mechanisms have been suggested for age-related changes previously observed in aggrecan structure. Interestingly, Sivan et al. proposed that, as a consequence of elevated proteases in ageing discs, proteolysis on the N-terminal end of aggrecan results in only the G1 domain remaining bound to the hyaluronan, which leads to the accumulation of unbound aggrecan fragments in the NP [33]. Additionally, aged aggrecan is known to accumulate AGEs on its lysine residues in vivo [37], and lysine residues within the G1 domain are critical for its hyaluronan interaction [33]. Collectively, these insights highlight the functional importance of this N-terminal protein region in aggrecan and may provide clues for the mechanisms (e.g., proteolysis or glycation) behind the tissue region-specific differences in peptide yield observed within this protein region between aged and young samples.

It is important to note that these displays of tissue region-specific modifications to protein structures were not limited to ECM proteins. For instance, similar peptide pattern differences were observed within the immune regulator complement factor H (*CFH*) (Figure S7), which also exhibited anterior inner AF-specific regional difference between young and aged samples, on the C-terminal side of its primary structure, and also tissue region-wide differences near its N-terminus. *CFH* plays a key role in regulating the progression of inflammation in age-related diseases such as vascular atherosclerosis [61] and macular degeneration [62]. The structural modifications observed here suggest that similar *CFH*-driven inflammation pathways may be involved in regions of the inner AF.

Collectively, these observations indicate not only the possibility of regional downstream consequences of ageing to major ECM components, on a molecular level, but also the potential of mechanisms acting exclusively within specific IVD regions.

## 3. Materials and Methods

### 3.1. Dataset Collection

In this study, young and aged IVD LC–MS/MS datasets were sourced from the Proteomics Identification Database (PRIDE)—Project ID PXD017740. These were originally generated for an earlier study which led to the spatiotemporal IVD proteomic resource DIPPER—http://www.sbms.hku.hk/dclab/DIPPER/ (last accessed 13 September 2021). For detailed methods of human tissue sourcing, sample preparation and MS, please refer to this original publication [13]. In brief, the datasets were generated by LC–MS/MS (data-dependant acquisition on an Orbitrap Fusion Lumos Tribrid Mass Spectrometer) from trypsin/LysC-digested whole protein samples extracted from 11 separate regions of IVDs—three discs (L3/4, L4/5 and L5/S1)—sourced from two male cadaveric human lumbar spines, one young (16-year-old) and one aged (59-year-old). Prior to LC–MS/MS, peptides were fractionated into four samples per IVD region by high-pH reversed-phase peptide fractionation and separately analysed. For this study, only 3 out of the 11 IVD regions available for analysis were evaluated by PLF: the anterior, left lateral and posterior inner AF/NPs (Figure 1a). PLF measures the relative difference in regional digestibility within proteins between two groups, and so it is important to note that the more preserved the native quaternary structure of the protein is, the more differences should be identifiable using this approach. Unfortunately, all protein extraction methods will inherently change the higher order structure of a protein. However, because all proteins in the samples tested were originally extracted using identical sample preparation methods [13], the regional differences measured are therefore a direct reflection of their native structure, prior to extraction. For this reason, PLF can be applied to historic datasets regardless of the extraction protocol used, as we have shown previously for ageing tendon [25].

It is important to highlight that the acquisition of non-degenerated human IVD tissue is challenging, and such samples from young and aged individuals are rare. Although we acknowledge that this detailed analysis was performed on one young and one aged individual only, this limitation is outweighed by the merits of the study, since our findings are based on human in vivo data and, therefore, of direct clinical relevance.

### 3.2. Peptide Identification

Raw MS spectrum files were converted to Mascot MGF files using RawConverter (Scripps Research Institute, La Jolla, CA, USA) [63], and peak spectra searched against the Uniprot human database (Swiss-Prot and TreEMBL; 2018) [64] using Mascot (Matrix Science, MA, USA). MS/MS ion searches were performed using the following parameters—instrument: ESI-TRAP; database: Swissprot_TreEMBL_2018_01; species: *Homo sapiens* (161,629 protein isoforms); fragment tolerance: 20 ppm; parent tolerance: 6 ppm (monoisotopic); fixed modifications: +58 Da on C (carboxymethyl); variable modifications: +16 Da on M (oxidation); digestion enzyme: trypsin; max missed cleavages: 2; peptide charge: 2+, 3+ and 4+. Mascot search results were exported as DAT files.

Scaffold 5 (Proteome Software, Portland, OR, USA) was then used to generate high-confidence PSMs by standard LFDR scoring. Mascot DAT files were imported into Scaffold 5, and individual fractionated peptide sample datasets were combined to form a single IVD region sample dataset using MudPIT (Stowers Institute, Kansas, MO, USA) [65]. Only peptides exclusive to their matched proteins and with a peptide probability of 95% minimum were used for PLF analysis, giving a low false discovery rate (FDR) of 0.6% per group (posterior, left lateral and anterior). Peptide FDR was calculated by the PeptideProphet algorithm (open source: http://peptideprophet.sourceforge.net/, accessed on 13 September 2021) [66] within Scaffold 5 using peptide probabilities designated by the Trans-Proteomic Pipeline. CSV files containing peptide lists were exported from Scaffold 5 for PLF analysis (Tables S1–S3).

### 3.3. Peptide Location Fingerprinting

Peptide lists were imported into our MPLF webtool (accessible at https://www.manchesterproteome.manchester.ac.uk/#/MPLF) (accessed on 13 September 2021), and three separate PLF analyses were performed on young vs. aged molecules for each IVD region (posterior, left lateral and anterior). As previously described [25] (Figure 1b), protein primary sequences were computationally divided into 50-aa-sized segments, and peptide sequences and their spectrum counts were mapped and summed within each segment. Sequences spanning two adjoining segments were counted in both. In order to identify differences in peptide yield along protein structures that are independent of differences in whole protein abundance, peptide counts in each 50-aa segment were normalised based on the experiment-wide (young and aged), median whole spectrum count of its corresponding protein. As a result of this protein-specific normalisation, each protein was treated as its own separate experiment during statistical comparison. Average, normalised peptide counts in each segment for the young group were subtracted from those in the aged group and divided by the segment aa length (50) to reveal regional fluctuations in peptide yield within the primary structures of proteins. Average, normalised peptide counts per protein segment were statistically compared between young and aged groups using unpaired, Bonferroni-corrected, repeated-measures ANOVAs (Tables S4–S6). Proteins which were only present in one group (young or aged) were excluded from the analysis. For more information on PLF and the MPLF webtool, please refer to our earlier publication [25].

### 3.4. Interaction Network Analysis

Exemplar proteins *COL1A2*, *PCOLCE*, *COL5A1*, *COL2A1* and aggrecan were used to construct an experimentally identified interaction network (Figure S4). ECM protein interactions were derived using Matrix DB [67], which were constructed and visualised using Cytoscape 3.4.0. (open source: https://cytoscape.org/, accessed on 13 September 2021) [68].

## 4. Conclusions

Although ageing and degeneration of IVDs are the result of genetics, environments and their interplays [5], this study focused predominantly on the identification of ECM proteins that may have accumulated modifications to structure over time. This was successfully enabled through the application of PLF to historic LC–MS/MS datasets generated from the inner AF of posterior, lateral and anterior regions of young and aged human IVDs. We demonstrated that, although many of these affected proteins were region-specific, several were identified in all three regions tested. Some of these shared proteins, such as *COL1A2* and *PCOLCE*, exhibited remarkably consistent age-associated differences in peptide yields along their protein structures between all tissue regions. This indicates the possibility that the same mechanisms of ageing may act on proteins across the entire tissue, leading to similar molecular-level consequences. However, several proteins identified, such as *COL5A1*, *COL2A1* and *ACAN*, also had lateral-specific differences in structure between aged and young samples, which indicates the possibility that some mechanisms acting on proteins may also be exclusive to certain tissue regions. This suggests that protein structures are potentially spatially and age-dependent in the IVD and that PLF is capable of detecting differences within localised tissue regions. This study highlights the merits of PLF as an interrogator of structural changes within proteins and potentially as a powerful tool for aiding biomarker discovery [25], which, in tandem with relative protein quantification (achieved in the DIPPER study [13]), allows for a more complete assessment of tissue proteostasis.

## Data Availability

The LC–MS/MS raw data were downloaded from the ProteomeXchange Consortium via the PRIDE repository; dataset identifier PXD017740. Interactive protein schematics showcasing the PLF analysis of these IVD datasets can be viewed (and the comparison datasets downloaded) via the MPLF webtool—https://www.manchesterproteome.manchester.ac.uk/#/MPLF by selecting the “Location Fingerprinter” tab (accessed on 13 September 2021). The DIPPER resource [13] is accessible at: http://www.sbms.hku.hk/dclab/DIPPER/ (accessed on 13 September 2021).

## Supplementary Materials

The following are available online at https://www.mdpi.com/article/10.3390/ijms221910408/s1.
