# Supplementary material for: Peptide Location Fingerprinting Reveals Tissue Region-Specific Differences in Protein Structures in an Ageing Human Organ
Source: Int J Mol Sci. 2021 Sep 27;22(19):10408. doi: 10.3390/ijms221910408 (PMC8509034; doi:10.3390/ijms221910408)
Supplement: Supplementary file 1 [file ijms-22-10408-s001.zip › ijms-1321445-supplementary.pdf]

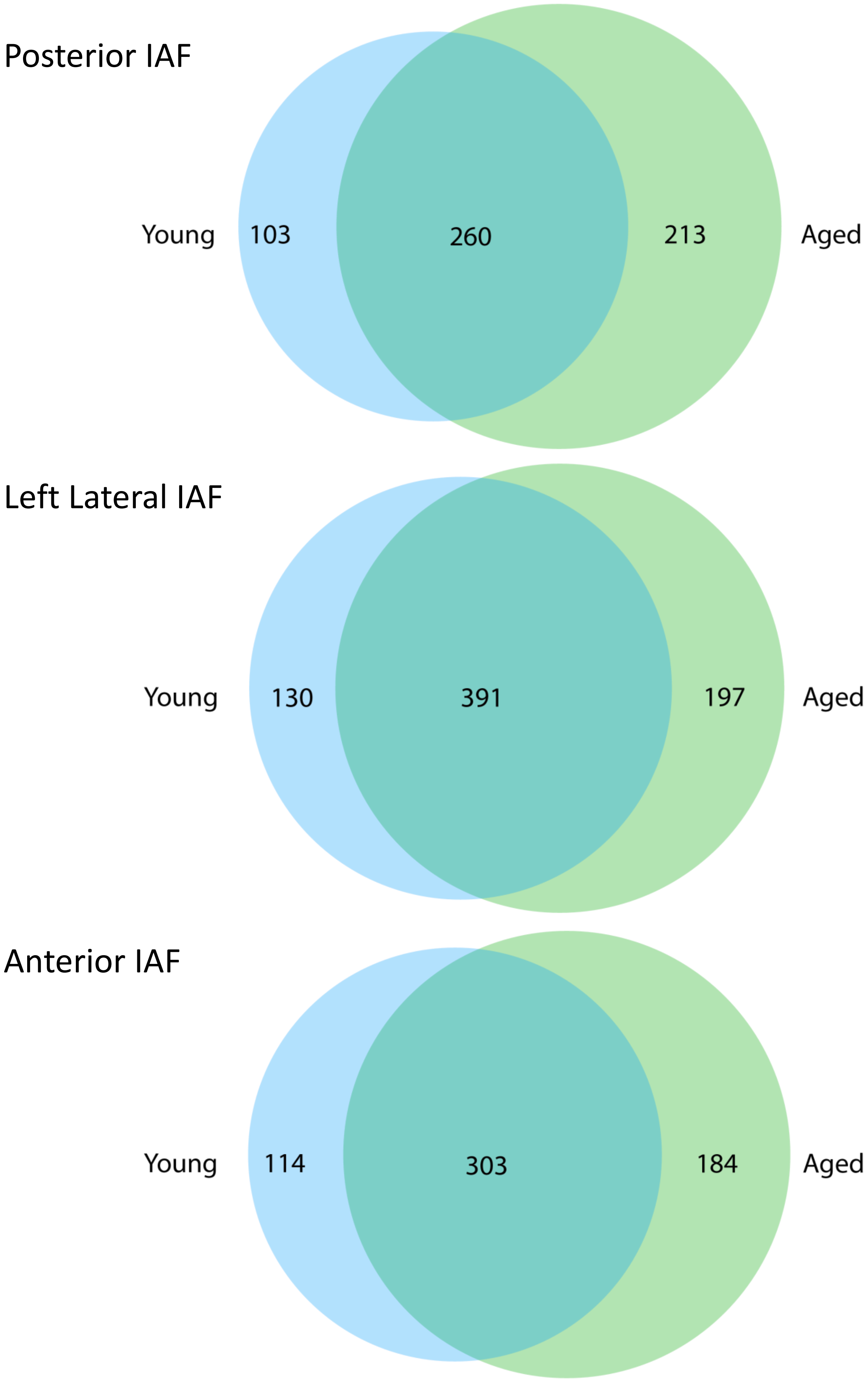

Figure S1. Number of proteins identified in young and aged samples from each IVD inner AF region by MS/MS peptide identification

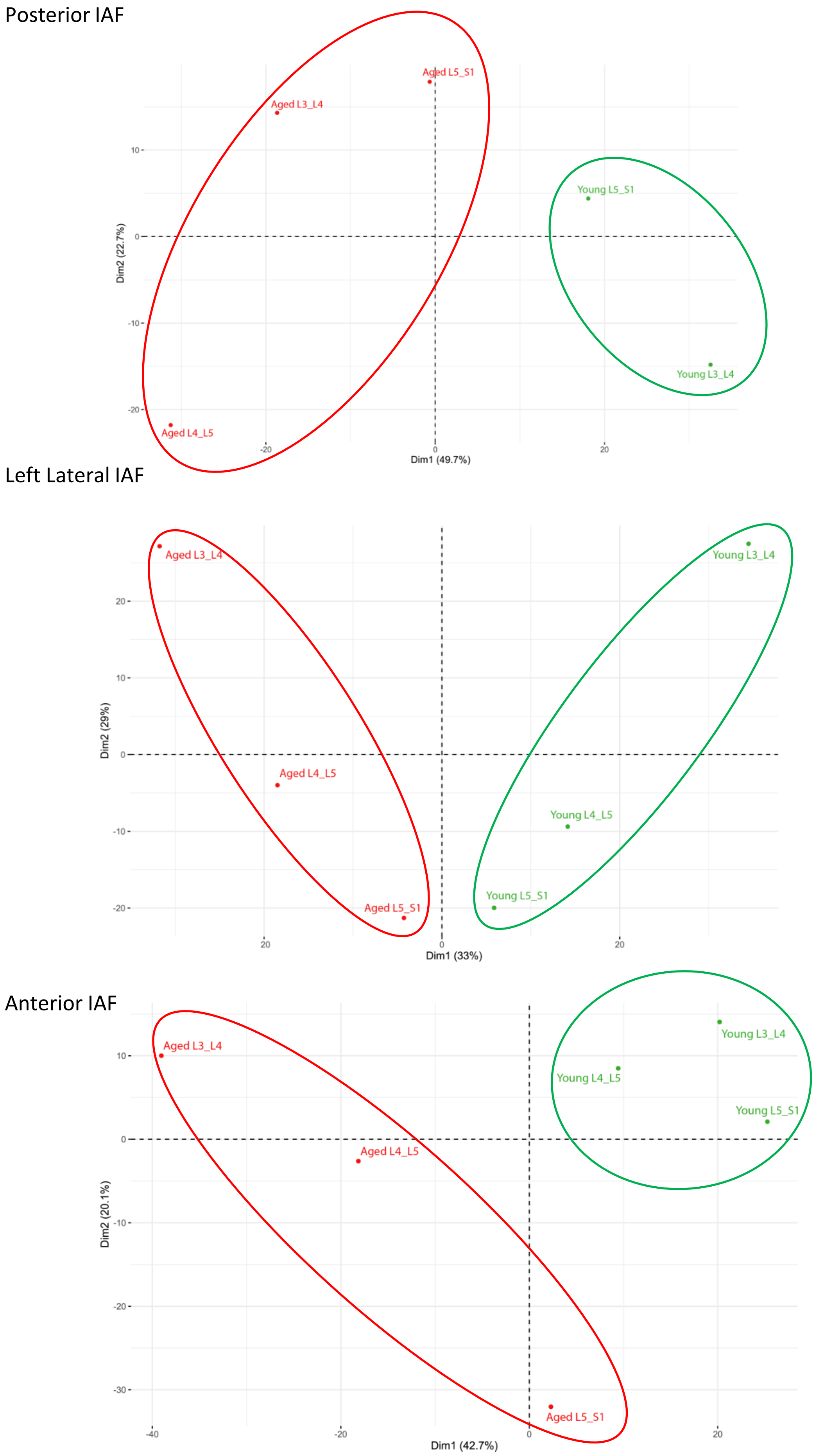

Figure S2. Principal component analyses of spectral count data used for peptide location fingerprinting. Aged (red) and young (green) data points separate into two distinct clusters for each IVD regions tested.

Figure S3. Potential biomarkers of inner AF ageing identified in the DIPPER study by label-free quantification (LFQ) of relative protein abundance and by peptide location fingerprinting (PLF; (Swiss-prot only). PLF identified 119 proteins that were unique to the approach, with age-associated significant differences in protein structure but not abundance

LFQ-identified biomarker candidates with differences whole protein relative abundance (DIPPER)

ACTA2, AFM, ALB, ALDOA, ANXA4, APOA4, APOD, APOH, ASPN, ATRN, AZGP1, C2, C6, C7, C8B, C9, CALM3, CDH1, CKM, COL11A2, CRYAB, CXCL12, CYTL1, DKK3, ECM1, EEF1A1, F2, FGB, FGG, FN1, HAPLN1, HIST1H1C, HP, HPX, HSPA1, HSPG2, HTRA1, HTRA3, IGHA1, IGHG1, IGK, IGKC, IGLL5, IL17B, ITIH1, ITIH2, ITIH4, KRT14, KRT19, KRT8, LECT1, LECT2, LGALS3, LOX, LOXL2, LYZ, MATN3, MFI2, P4HB, PGAM1, PGK1, PLG, POSTN, PPIA, PPIB, PRDX1, PTRF, RBP4, RNASE3, S100A1, SCUBE1, SERPINC1, SERPIND1, SERPINF1, SLPI, SSC5D, TF, TIMP1, TIMP3, TKT, TNFRSF11B, TNXB, TPI1, UGP2, VIM

Biomarker candidates identified by PLF with differences in protein structure

ACAN, ACTB, ACTG1, AEBP1, AGT, ALDOC, AMBP, APOE, ATP1A1, B3GNT7, C1R, CALR, CAVIN, , CCN2, CFH, CHRD, CHST14, CHST3, CILP, CILP2, CLEC11A, COL18A1, COL1A1, COL7A1, COL9A1, COL9A2, COMP, CP, CRTAC1, DAG1, DNAJC3, EDIL3, ENO2, FBLN1, FBLN2, FLT1, FMOD, FSTL1, FTH1, GALNT18, GALNT2, GANAB, GDF5, GPX3, H1-2, H1-4, H2AC16, H2BC11, H2BC17, H2BC21, H2BC3, H4-16, HBB, HBG1, HBG2, HEL70, HHIPL1, HRG, HSP90B1, HSPA5, IGFBP7, INHBA, IQGAP1, ISLR, KRT5, LAMB2, LDHB, LOXL3, LRP1, LTBP2, LUM, MATN4, MFGE8, MMP3, MSN, MXRA5, MYH7, NUCB1, OAF, OMD, PAM, PDGFRL, PDIA3, PDIA4, PFKP, PLEC, PLOD1, PLOD2, PLTP, PLXDC2, PRDX2, PRDX4, PRDX5, PRDX6, PRELP, PRKCSH, PROS1, PXDN, RPS27A, SCIN, SEMA3A, SERPINA3, SERPINE2, SERPING1, SLIT3, SMOC1, SNED1, SOD3, SPP1, SRPX2, TALDO1, TGM2, THBS1, THBS2, TIMP2, TNFAIP6, TUBA1A, UBA52, VASN, VWA1

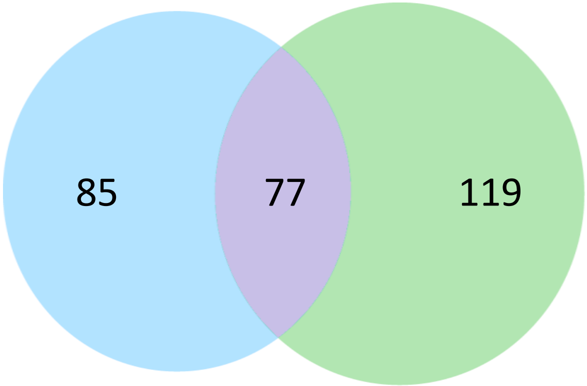

Biomarker candidates identified by both LFQ and PLF

A2M, ABI3BP AHNAK, ANG, ANGPTL2, ANXA1, ANXA2, ANXA5, ANXA6, APOA1, BGN, C3, C5, CALU, CCDC80, CD109, CHAD, CHADL, CHRDL2, CLEC3A, CLSTN1, CLU, COL11A1, COL12A1, COL14A1, COL15A1, COL1A2, COL2A1, COL3A1, COL5A1, COL5A2, COL6A1, COL6A2, COL6A3, CSPG4, DSC3, ECM2, EMILIN1, EMILIN3, ENO1, ENPP2, F13A1, FBN1, FGA, FNDC1, FRZB, GAPDH, HHIPL2, ITIH5, LAMA5, LDHA, LMNA, MATN2, NID2, ORM1, PCOLCE, PCOLCE2, PEBP1, PKM, PRG4, PYGB, QSOX1, RCN3, RNASE4, SEMA3C, SERPINA1, SERPINA5, SERPINE1, SMOC2, THBS3, THBS4, TNC, TUBA1B, TUBB4B, VCAN, XYLT1, YWHAE

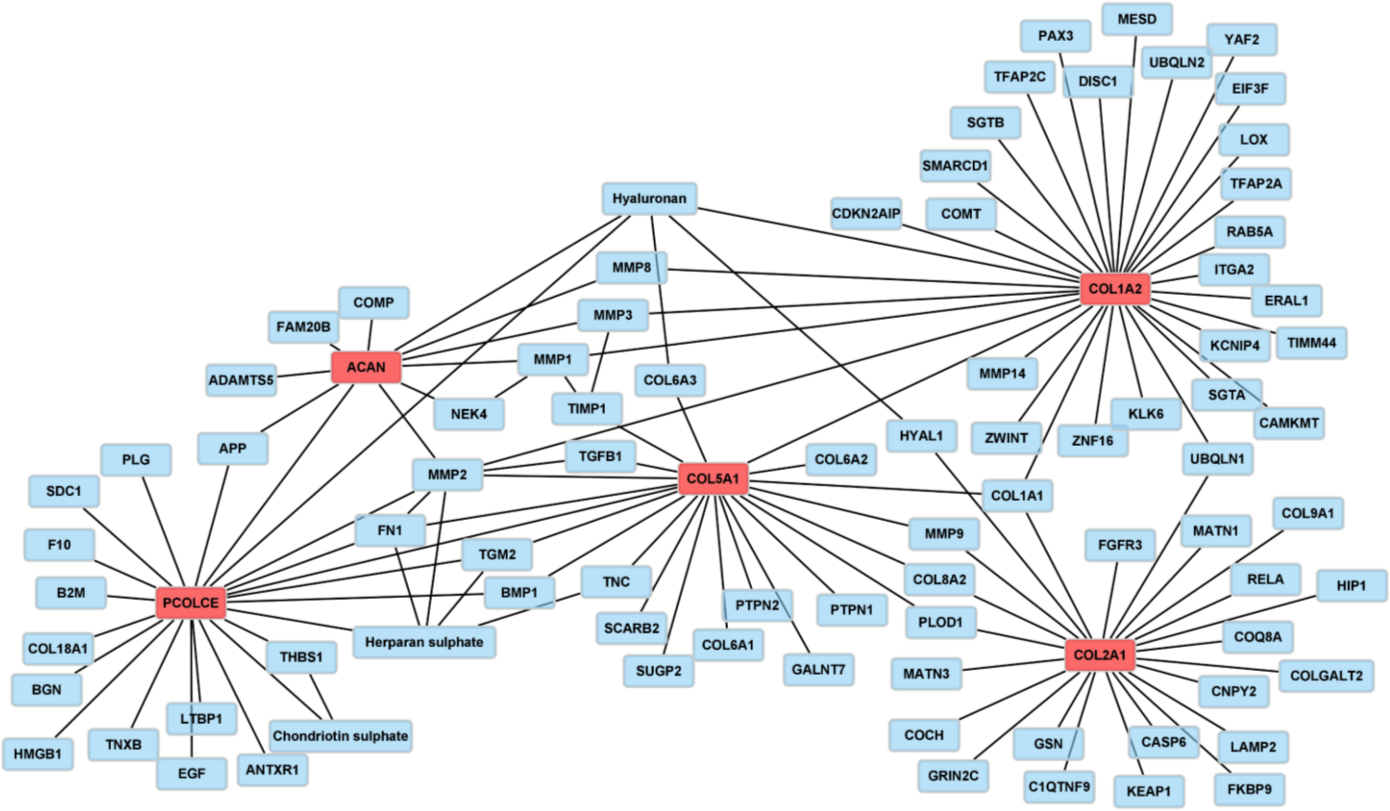

Figure S4. Exemplar ECM proteins identified with age-dependent, structural modifications (red) and their direct experimental interactors (blue). Interactions were curated from the IntAct and MatrixDB databases.

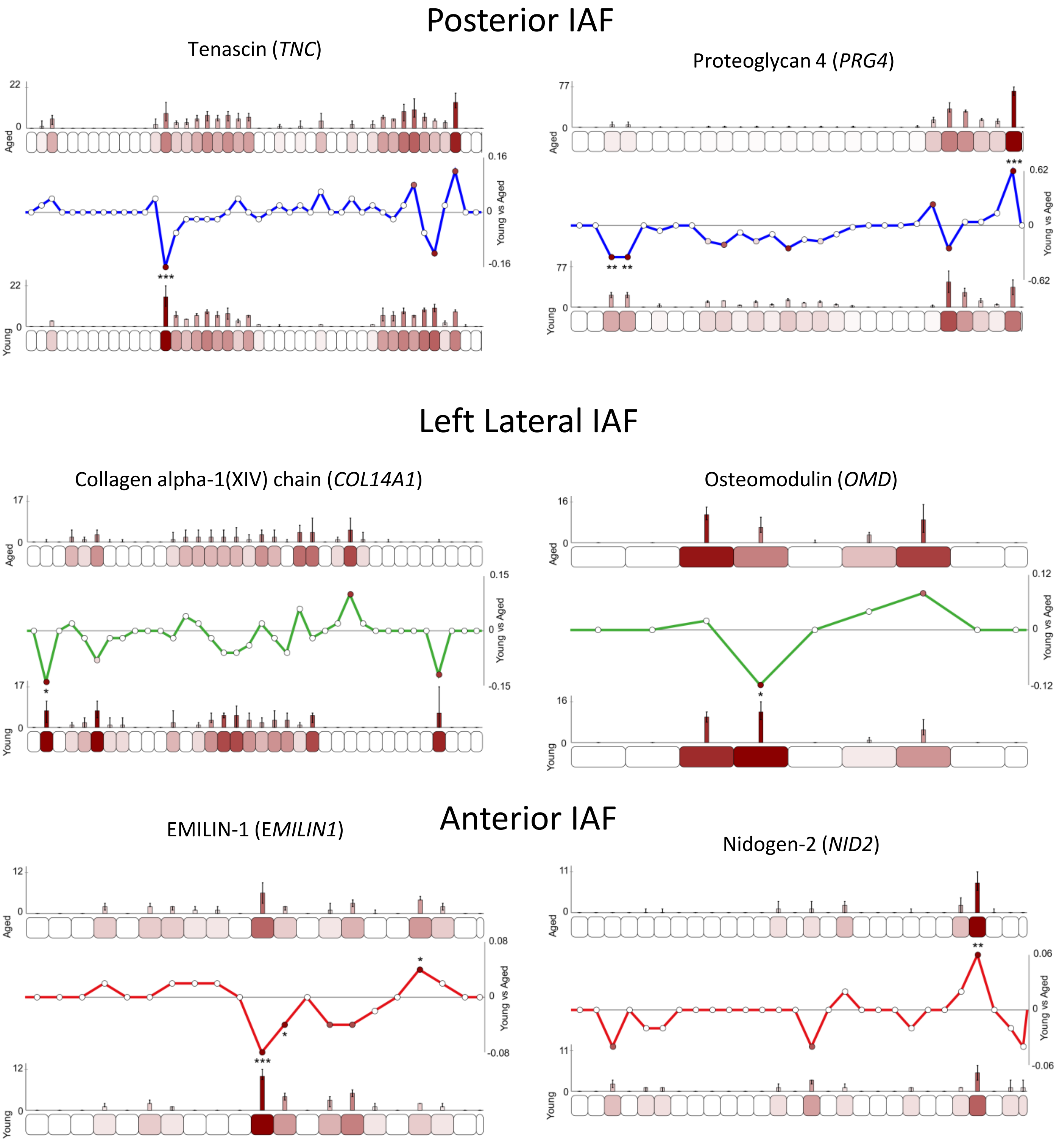

Figure S5. Exemplar ECM proteins exhibiting IVD region-specific, age-dependant differences in peptide yield patterns. PSMs were summed in each 50 aa protein segment, normalised and averaged for young and aged groups (bar graphs = average, normalised PSMs, error bars = standard deviation). Average PSMs per segment in young were subtracted by those in aged and divided by segment aa length (50) to reveal fluctuations in peptide yield along protein structures (line graph y axes = aged – young PSM counts / segment length). Several regions along the structures of these proteins exhibited significant differences in peptide yields between young and aged (*, p ≤ 0.05; ** p ≤ 0.01; ***, p ≤ 0.001, Bonferroni-corrected, repeated measures ANOVA).

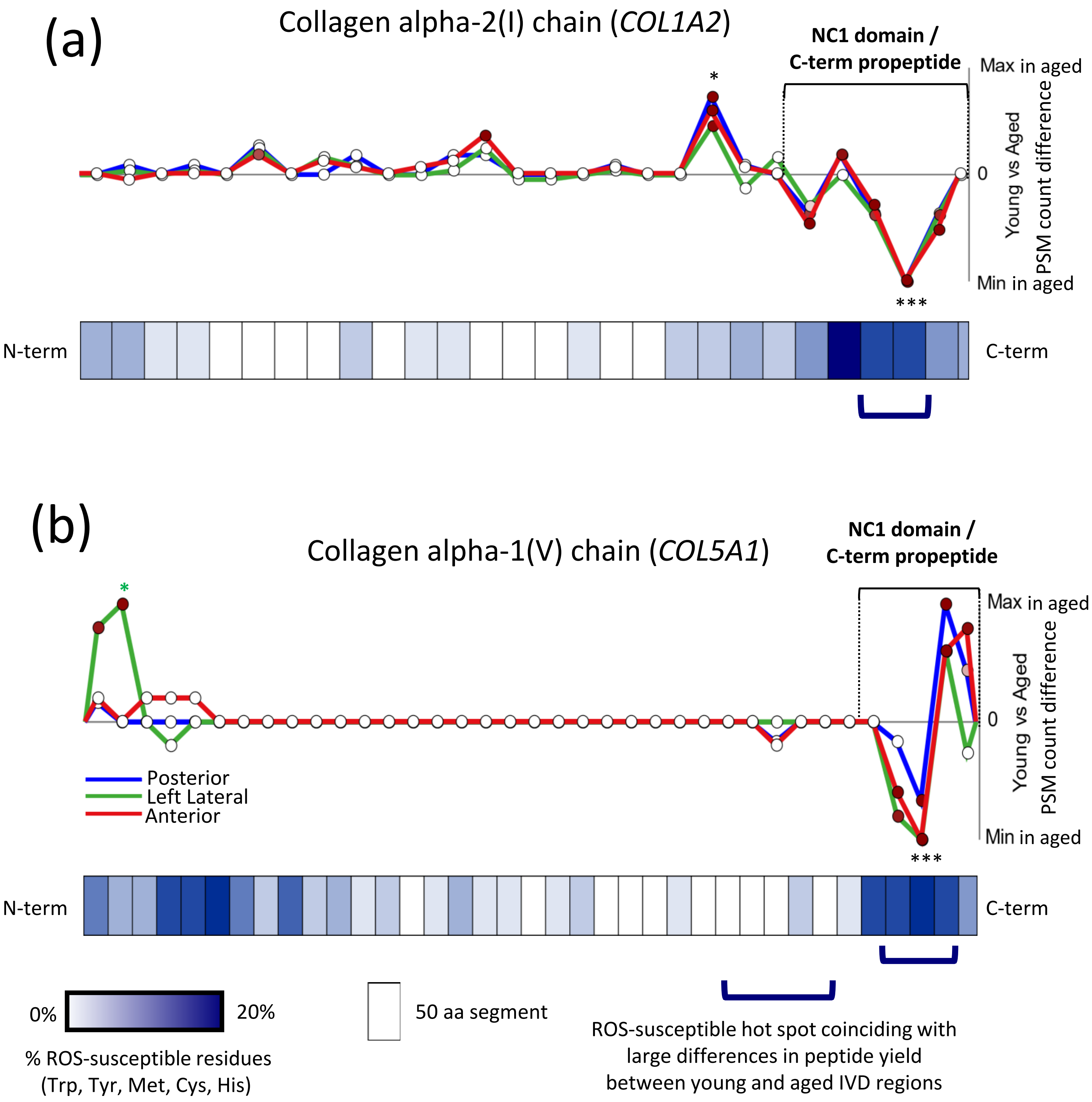

Figure S6. Tissue region-conserved, age-dependant peptide yield differences along the structures of *COL1A2* and *COL5A1* coincide with protein regions enriched in ROS-susceptible aa residues. The percentage content of ROS-susceptible residues (Trp, Tyr, Met, Cys, His) were calculated within 50 aa sized-segments for each protein using our MPSC webtool (accessible at https://www.manchesterproteome.manchester.ac.uk/#/MPSC) and displayed as a heat map with composite line graphs of *COL1A2* and *COL5A1* (from Figs. 3Aii and 5Aii) showing peptide yield differences between young and aged aligned. (a) Two segments (second and third to last) on the C terminal end of *COL1A2*, which had high contents of ROS-sensitive residues (14% in both) also exhibited lower peptide yields in aged than in young (significant for the second to last segment). (b) Similarly, three segments on the C-terminal end of *COL5A1* which were enriched ROS-susceptible residues (second [14%], third [16%] and fourth [14%] from the C-terminus) exhibited lower peptide yields in aged than in young.

Complement factor H (*CFH*)

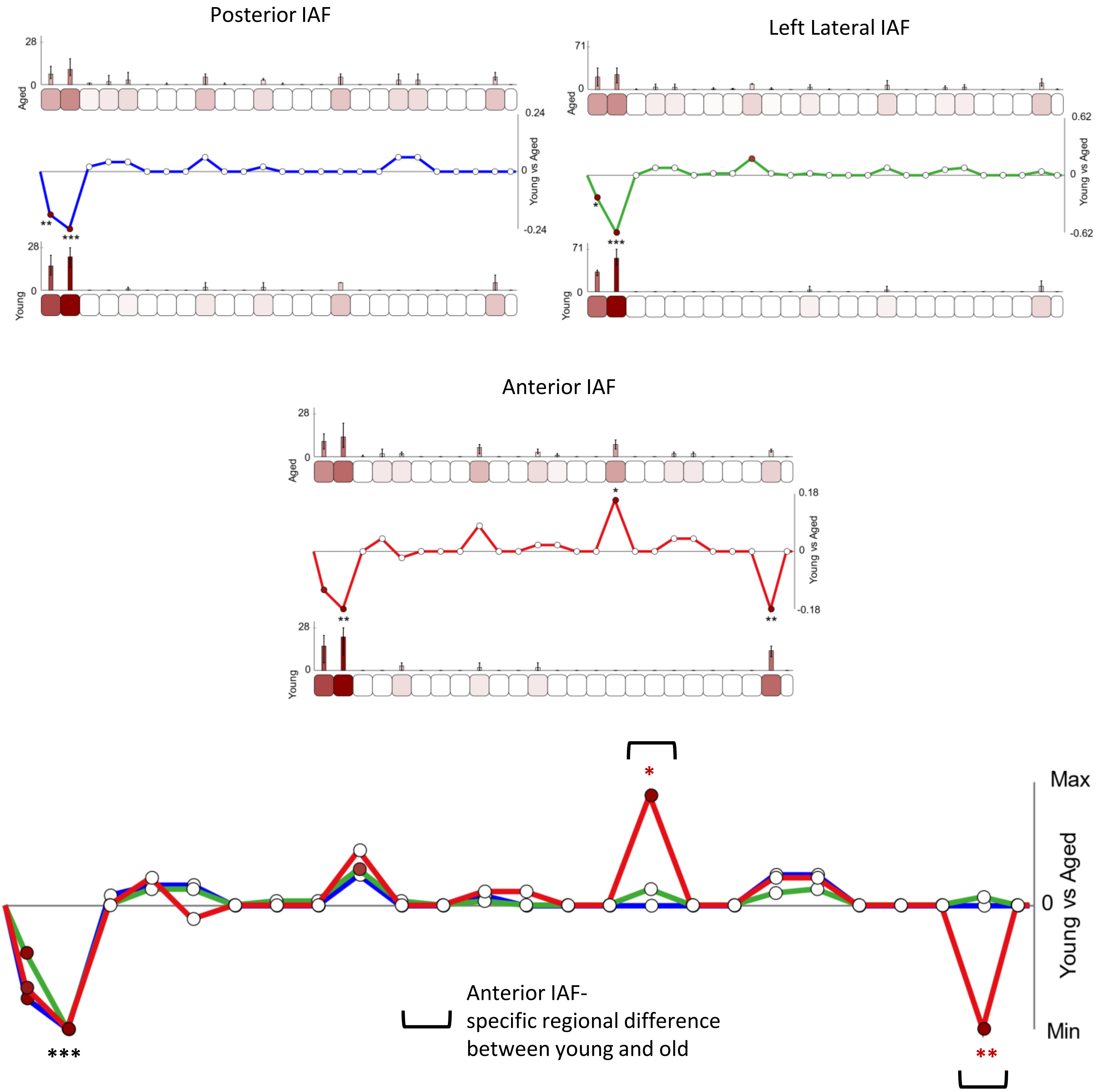

Figure S7. Complement factor H (*CFH*) exhibited anterior, inner AF-specific differences in peptide yield not seen in posterior or anterior IVD regions. PSMs were summed in each 50 aa protein segment, normalised and averaged for young and aged groups (bar graphs = average, normalised PSMs, error bars = standard deviation). Average PSMs per segment in young were subtracted by those in aged and divided by segment aa length (50) to reveal fluctuations in peptide yield along protein structures (line graph y axes = aged – young PSM counts / segment length, normalised between each region in composite graph; *, p ≤ 0.05; ** p ≤ 0.01; ***, p ≤ 0.001, Bonferroni-corrected, repeated measures ANOVA). One region, on the N-terminal end of CFH, exhibited significantly lower peptide yields in aged than in young for all three IVD regions. However, two anterior-specific significant difference in peptide yield were also observed near the central portion of the protein and near the C-terminal end.
